# New Remedies in Veterinary Medicine*Read before the German Veterinary Medical Association of New York and Vicinity.

**Published:** 1894-01

**Authors:** Fred. W. Turner


THE JOURNAL

OF

COMPARATIVE MEDICINE AND

VETERINARY ARCHIVES.

VOL. XV. JANUARY, 1894. NO. 1.

### NEW REMEDIES IN VETERINARY MEDICINE.*

By FRED. W. TURNER, PH.G., D.V.S.

The restless, progressive tendency of modern times is perhaps nowhere more conspicuous than in pharmacology, and especially in those drugs of a synthetical character, which have become one of the features of our time. The zeal and untiring energy of the old alchemists in their search for the philosopher's stone and the elixir of life are reproduced to-day in the eager quest of their scientific descendents for artificial alkaloids. Some of these new medical agents have won the favor of the medical profession, and have secured a permanent place in the pharmacopœias, while others are still on trial passing through their period of probation. The literature of substances produced at different times by various manufacturers and examined by pharmacologists and chemists in all parts of the civilized world is widely scattered and not easy to come at. For this and other reasons it has become exceedingly difficult, in fact almost an impossibility, for the busy medical practitioner to maintain his acquaintance with the properties and uses of therapeutical novelties continually brought to his notice in the scientific press of the country. Since the time when the modern chemist became fired with the ambition to win fame and wealth at one stroke by the synthesis of quinine, the number of remedies turned out yearly from the chemical laboratory has gone on steadily increasing, and if the original aim of the work is still unaccomplished—as the process of Grimmaux and Arnaud is but a partial solution of the problem—yet among the very considerable number of compounds produced, some have been found capable of

* Read before the German Veterinary Medical Association of New York and Vicinity.

replacing the natural alkaloid in many cases, while in others they seem to be even superior to it in the therapeutical activity, reliability or safety. A great number of those "new remedies" are of no use in veterinary practice in general, owing to their great cost and mode of administration, and I will only mention those which are known to have been applied successfully by veterinarians either in surgery or medicine.

ARISTOL.—A brownish red powder, insoluble in water and glycerine; contains about 46 per cent. of iodine; slightly soluble in alcohol, more so in ether. It is prepared by the decomposition of a solution of iodine in iodide of potash by an alcoholic solution of thymol. It is decomposed by light and heat. Aristol was introduced as a substitute for iodoform, having the advantage of being odorless. It may be employed in all cases where iodoform is indicated, and in the same manner, either as a powder, ointment or collodion.

CHLORALAMIDE.—Prepared by the interaction of chloral (not chloral hydrate) and formamide. It consists of lustrous colorless crystals with a somewhat bitter taste, soluble in 20 parts of water and 2 parts of alcohol. It was introduced as a substitute for chloral hydrate, being safer to give to patients suffering from any cardiac trouble, as there are no unpleasant or dangerous by or after effects. The dose is the same as in chloral hydrate, and its use is indicated wherever chloral is employed.

DITHIOSALICYLIC ACID—a substance prepared by heating equal molecular weights of salicylic acid and sulphur chloride to 150 degrees Celsius, forming a yellow resinous mass, which is dissolved in water containing soda, and the acid is precipitated by addition of hydrochloric acid. The acid itself has been hardly ever used in medicine, but the sodium salt is—sodium dithiosalicylate. This salt has been highly recommended in foot and mouth disease in cattle, being employed as lotion or compresses in 2½ to 5 per cent. solution. Strikingly beneficial results are reported, and the drug seems worthy of further trial.

DIURETIN—prepared by the interaction of sodio-theobromine and sodium salicylate. It is claimed for this remedy, that if the stomach and intestines are kept in an alkaline condition, that it will prove of great benefit in cases of azoturia in doses of two drachms every four to six hours.

ETHYL BROMIDE OR AETHER BROMATUS—a colorless, limpid, inflammable liquid with a sweet chloroformic odor and burning taste, is very largely used as a general anæsthetic in minor surgery. The narcocis is produced in from one-half to one minute, and lasts only a few minutes, unless fresh quantities are administered.

Exalgine—a substance occurring in beautiful needle-like crystals, has been highly praised for the treatment of chorea or St. Vitus' dance in dogs—to be given in doses from one to two grains three times daily.

Ichthyol—a product won by dry distillation from a bituminous mineral, found in Tyrol in Southern Europe, supposed to be derived principally from fish. Ichthyol is clear, thick brown liquid of a smoky odor and taste. Soluble in water, alcohol, ether and fats. It may be given internally or externally without the least danger. It contains about 15 per cent. of sulphur. The action of ichthyol when applied in medicine. depends chiefly upon three factors: First, its reducing properties; second, its vascular contractile effects; third, its antiseptic action. From this combination of properties, ichthyol has proved useful as an antiphlogistic, an alterative, an anodyne, resolvent and astringent. Its restraining influence upon the development of bacteria has repeatedly been proved and confirmed by practical experience, its peculiar virtues are largely ascribed to the proportion of sulphur it contains. It was first introduced by Dr. Unna as a panacea for skin diseases only, but since then the list of affections in which ichthyol has been successfully used has grown to a great extent. Internally, one ounce may be given in twenty-four hours.

Lanolin—prepared from the crude wool fat by emulsification with hydrate or carbonate of the alkalies, forming a whitish substance, free from odor, insoluble in water, more soluble in alcohol, readily soluble in ether and benzine. Three factors have been of great importance in determining the permanent retention of the substance as a vehicle for the use of external medication to the skin: First, its resistance to the agents which produce rancidity; second, its property of taking up a great quantity of water without losing its ointment-like consistence; third, the completeness with which it is absorbed by the skin. To these must be added that lanolin is perfectly free from any irritating constituents. When lanolin is rubbed upon the skin it is rapidly taken up by the membrane, and any medicaments mixed with it are at the same time absorbed. The advantages of lanolin over other ointment bases are so marked that it has been almost exclusively and universally adopted for the application of the newer remedies in ointment form. Even acids, chloride of calcium, peroxide of hydrogen, sulphurous acids and other substances, of which heretofore ointments could not be usefully prepared, may be successfully applied with lanolin. The sole practical objection to the use of lanolin for ointments containing only solid ingredients, is its stickiness, and this is readily overcome by mixing it with one-third of its weight of vaseline. This diluted vaseline meets all the requirements of a good ointment base, and its value has been confirmed by the experience of numerous medical authorities.

Piperazine—is formed by the action of ammonia on ethylene bromide or chloride. It is readily soluble in water, and may be employed hypodermically. The most valuable chemical property of piperazine is its power of forming a readily soluble compound with uric acid. There seems to be a great future for this remedy, but it needs more investigation. As a solvent for uric acid and urate concretions, it is far superior to all known medicaments. A very marked relief is afforded by the remedy in the pruritus of the uric acid diathesis, due to the irritation of imperfect nitrogenous elimination. This drug ought to do the work in azoturia to perfection, for it will render soluble twelve times as much of uric acid deposits as any of the lithia salts.

It may be given in drachm doses, repeated at intervals from four to six hours. Being without caustic action upon mucous membrane. aqueous solutions of piperazine (2 to 4 per cent.) may be directly introduced into the bladder for the purpose of dissolving vesical stones.

Salol—or phenyl salicylate, a preparation obtained by prolonged heating of salicylate of soda and phosphoric oxichloride, until reaction has taken place. It forms a white crystalline tasteless powder of transparent crystals, being insoluble in water. Salol was prepared and introduced into medicine after physiological examination, as the result of researches with the view of discovering a compound of salicylic acid which would be free from disadvantages of most salicylates.

Experiments have shown that salol possesses antiseptic, antipyretic and anti-rheumatic properties. At the same time it was found that the remedy, being insoluble, passes unabsorbed and unchanged through the stomach. and is only decomposed into salicylic acid and phenol compounds by the alkaline juices of the intestines. Due to this intestinal dissociation its particular virtues are largely due, as by its means local antisepsis becomes possible in such affections as acute diarrhœa, dysentery, or cholera. Eight grains of salol and three grains of bismuth-salicylate, given every three hours has been a favorite prescription for cases of Asiatic cholera.

THIOL—Prepared from brown-colored paraffine or gas oils, which are treated with sulphur at a high temperature, the unsaturated hydrocarbons which are attacked alone by the sulphur, are extracted by suitable solvents from admixture with saturated hydrocarbons ; by the action of concentrated sulphuric acid under artificial cooling, thiol is obtained. Liquid thiol is a thin brownish-black liquid, neutral, with a feeble bituminous odor, forms clear solutions with water, only partly soluble in alcohol and ether.

Thiol was introduced in the materia medica in 1888, as an additional remedy against the many forms of skin diseases which come under treatment. The literature on thiol contains reports of its successful use in eczema, acne, sycosis, erythema, erysipelas, lymphangitis, and general eczematous affections, also acute inflammatory processes of the skin and tissues, acute infiltrations of joints, œdema, contusions and subcutaneous hæmorrhage. Thiol may be applied as a powder, a 5 per cent. collodion made from the thiolum siccum, aqueous or glycerine solutions from 10 to 50 per cent. Very satisfactory results were also obtained by the treatment of wounds, whether superficial or penetrating, and of burns with dry thiol powder, used as a dusting powder.

According to numerous observations the effect of the powder was displayed in converting serum (the contents of the blisters caused by burns) into a solid more or less transparent, gelatinous mass, or scab, under which the process of healing went rapidly forward. Consideration of the action of thiol upon skin diseases and injuries outlined above shows that the principal physiological effects of its external application may be divided into four classes, namely:

1. A drying or desiccating property.
2. A muscular contractile property.
3. An antiseptic action.
4. A keroplastic or horn-forming tissue.

A careful examination of the evidence accumulated in medical literature, shows that thiol is a valuable enrichment to materia medica ; it brings into prominence certain facts in connection with its characters and actions.which have been followed by beneficial results, and not only so, but, in many cases, by results which the ordinary or routine remedies were powerless to bring about.

CREOLINE—is a by-product in the manufacture of carbolic acid. It is prepared from coal tar creosote, after the complete removal of carbolic acid, by the addition of resin and caustic soda. Creoline is a dark brown, syrupy, tar-like fluid, with a smoky odor similar to that of tar, and an aromatic, subsequently burning taste. Dropped into water, it at first forms whitish clouds, which soon coalesce into a milky, uniform emulsion, slightly alkaline in reaction. The limit of perfect emulsion in water is reached at 2½ per cent. Creoline is an antiseptic and disinfectant of the first rank. According to the bacteriological investigations of Prof. von Esmarch, it acts decidedly more powerfully than carbolic acid on pus micrococci, on typhus bacilli, and on cholera bacilli. A 1 to 1000 solution kills the cholera bacilli in ten minutes; a 5 to 1000 solution in one minute, whereas it takes a 1 to 1000 solution of carbolic acid four days to do the same. The typhus bacilli are checked in their formation by a 1 to 1000 solution of creoline distinctly already in one hour. A 1 to 1000 solution of carbolic acid exerts no restricting influence on their formation even after 22 days. The pus bacilli are distinctly hindered in their growth in one hour and are killed in four days, while carbolic acid fails completely to produce any effect in four days. Creoline deserves to be urgently recommended on account of its power of destroying and checking the formation of bacteria; on account of its absolute freedom from poisonous properties, its innocuousness and its cheapness. Administered internally, creoline is non-poisonous, and is not caustic even in undiluted form. It diminishes putrefaction and the formation of gases in the intestinal tract even in small doses. In large doses even the feces lose their bad odor. Creoline has been used to some extent in veterinary practice in Europe, it is a very efficient and at the same time non-poisonous antiparasitic, much preferable to carbolic acid, tobacco, arsenic and mercurial preparations in the treatment of mange. A good embrocation is made by equal parts of creoline and green soap, adding from 3 to 10 per cent. of alcohol according to the severity of the disease, to be applied once a day. The same liniment will answer in the treatment of mange of sheep, followed by a creoline bath of 2½ per cent. in strength. Creoline baths are preferable to others on account of being non-poisonous, easily prepared and cheap, they do not injure the fleece as tobacco, arsenic and others always do. Large doses may be given to animals internally without producing any toxic effects or symptoms whatsoever. Creoline is a reliable and rapid deodorant in the often extremely offensive ulceration of somatitis in dogs. As a disinfectant for dog kennels and stables a half per cent. solution generally gives satisfaction; its volatility gives it an advantage over corrosive sublimate. In the open treatment of wounds of domestic animals the following mixture has proved very serviceable: From 2½ to 5 parts of creoline to 100 parts of powdered boric acid are intimately rubbed together and used as a dressing powder to the wounds, which are previously cleaned by a 1 per cent. solution of creoline in water. In erysipelas of the legs of horses very good results have been obtained by rubbing the affected limbs once a day with a 5 per cent. alcoholic solution of creoline and then applying a linen bandage uniformly. Against worms in horses it is internally administered on an empty stomach, the dose given is 1½ oz. diluted in one quart of water. For killing lice or flees on domestic animals a 3 per cent. solution in water will give good results. Diphtheria in fowl has been successfully treated by painting the throat with a warm solution of one teaspoonful to a cup of water twice a day, and also giving of the same solution a teaspoonful, 2 a day, internally.

PYOKTANIN—English pus killer—is the name given to that group of colorific substances which are found most efficacious of all the pigments in regard to their bactericidal action. This group of pigments is so far represented in the materia medica by the following three varieties:

*1. Blue Pyoktanin*, the kind always meant and understood by the term "Pyoktanin," unless denominated to the contrary.

*2. Yellow Pyoktanin*, often preferred in ophthalmic and cutaneous diseases on account of its milder action, and

*3. Ethyl Pyoktanin*, the most permeant and powerful bactericide of the three.

Pyoktanin is derived from coal tar, an antiseptic, a true but harmless therapeutical disinfectant, an absolutely sure and yet perfectly safe bactericide, eminently adapted for permeation through animal tissues and fluids in the living body. It is devoid of any injurious effect on the human or animal economy or organism; certainly and utterly destructive of all bacteria or other micro-organisms absorbing it; rapidly diffusible in, and permeant of, both healthy and diseased animal tissues or fluids, and perfectly penetrant through the enveloping membranes and internal substance of all bacterial colonies harbored by them. These qualities of Pyoktanin make it not only the best antiseptic or germ arrestor now known to medical science, but also a practical medicinal disinfectant or germ destroyer in the living body. No other substance has so far been ascertained to possess the qualities above enumerated as pertaining to Pyoktanin, qualities essential to the efficacious and safe disinfection of all purulent, gangrenous, septic, syphilitic, tubercular or other micro-parasitic morbid processes.

Dr. Mehrdorf reports of 1293 cases of foot and mouth disease of all conceivable degrees of severity and in all stages. Of these animals treated, 1261 were cows, 28 hogs, and 4 goats. In accordance with directions, a 1 to 100 solution of pyoktanin was first employed, then a 1 to 500 one; but observations proved that still weaker solutions had just as favorable a result; a 1 to 1000 solution of pyoktanin was subsequently employed exclusively. After cleaning the cutaneous tissues about the hoofs affected with the specific inflammation, cloven feet, balls and surrounding parts, they were painted twice daily with a 1 to 1000 solution. In a few exceptional cases, where a gangrenous mortification of the cutaneous structures had set in, a firm antiseptic dressing was applied, so as to immobilize the pad of oakum, saturated with pyoktanin solution, which was placed between the hoofs. To subdue the disease about the mouth, lips and nostrils, these parts were thoroughly brushed over with the solution twice a day. In very recent cases almost complete restoration often followed the application of pyoktanin in even 24 hours, but in most cases after 48 hours. The inflammation in the mouth made no progress, on the contrary, it generally disappeared entirely, and the mucous discharge ceased. In the later stages of the disease, when there are extensive excoriations and ulcers in the mucous membrane and skin of the affected parts, the success is not so prompt, but it is nevertheless a striking one. The advantages of pyoktanin in the treatment of foot and mouth disease are essentially as follows:

1.—Loss of animals can be almost entirely avoided by its timely and proper use.

2.—Nutrition of the affected animals is but very slightly or not at all impaired during the treatment.

3.—The loss of milk is only of a few days' duration, and of no consequence; if the animals are properly fed, the lacteal secretions soon regain their former standard.

4.—The disease assumes a mild character and runs a favorable course.

5.—Working animals, such as oxen, can be used again in a short time.

Some authorities regard the intense staining power of pkoktanin as a drawback, but this property should be considered as a desideratum, because it enables the surgeon to see whether or not the remedy has been applied, and also how thoroughly. Pyoktanin is also adapted for the treatment of fresh wounds as they originate under the surgeon's knife, as well as for old and neglected wounds of an angry nature. Common to all wounds treated with pyoktanin seems to be a very striking limitation of the secretion from the wound, no matter what the previous appearance and character of the secretions have been. In ulcers of the cornea in the course of distemper in dogs a 1 to 5000 solution of pyoktanin has been used with success, effecting a rapid and undisturbed cure. Favorable results may be obtained from the use of pyoktanin in purulent inflammations of the skin, so frequent in dogs, which owing to the continual irritation, only slight originally, soon develop into extensive suppurations, and need attention in their early stages. There is one restriction to its use, in white-haired animals, we must consider beforehand whether it is permissible to impart a blue color to the hair for some time.

